# Preparation, Physicochemical Properties and Stability of Anthocyanin Nanoliposomes Before and After Double-Layer Modification Using Synanthrin and Pea Protein Isolate

**DOI:** 10.3390/molecules30142892

**Published:** 2025-07-08

**Authors:** Lianlian Zhang, Aniya, Shengping Xing, Jing Li, Ying Liu, Chaozhi Li, Jianhang Zhu, Yan Li, Xiaoji Fu

**Affiliations:** 1 National Key Laboratory of Food Science and Resource Mining, Nanchang University, Nanchang 330077, China; zll2422149552@163.com; 2 Institute of Agricultural Products Processing, Jiangxi Academy of Agricultural Sciences, Nanchang 330200, China; any2014@foxmail.com (A.); xsp_3000@126.com (S.X.); lijing11311@163.com (J.L.); yingliu1902@163.com (Y.L.); george3811@foxmail.com (C.L.); 18970056428@163.com (J.Z.)

**Keywords:** anthocyanin, pea protein isolate, synanthrin, nano-liposome, in vitro digestion, storage stability

## Abstract

Anthocyanins (ACNs), characterized by their polyhydroxy structures, exhibit high susceptibility to external environmental factors, which significantly limits their application in the food and industrial sectors. To enhance the stability of anthocyanins, anthocyanin nanoliposomes (ACN-NLs) were developed, with encapsulation efficiency, particle size and zeta potential serving as key evaluation parameters. Furthermore, through layer-by-layer self-assembly and electrostatic interactions, ACN-NLs were modified using synanthrin (SY) and pea protein isolate (PPI). Consequently, PPI-modified ACN-NLs (PPI-ACN-NLs) and SY-PPI-modified ACN-NLs (SY-PPI-ACN-NLs) were successfully synthesized. In this study, the structural characteristics of liposomes were investigated using X-ray diffraction (XRD), their in vitro digestibility was evaluated, and their stability under different temperatures, light conditions, and simulated food system conditions was assessed. The results demonstrated that when the mass ratio of soybean lecithin to cholesterol, soybean lecithin to anhydrous ethanol, and drug-to-lipid ratio were set at 5:1, 3:100, and 3:10, respectively, with an ACN concentration of 4 mg/mL, a pea protein solution with pH 3.0, a PPI concentration of 10 mg/mL, and an SY concentration of 8 mg/mL, the prepared ACN-NLs, PPI-ACN-NLs, and SY-PPI-ACN-NLs exhibited optimal performance. Their respective encapsulation efficiencies were 52.59 ± 0.24%, 83.80 ± 0.43%, and 90.38 ± 0.24%; average particle sizes were 134.60 ± 0.76 nm, 213.20 ± 0.41 nm, and 246.60 ± 0.24 nm zeta potentials were −32.4 ± 0.75 mV, −27.46 ± 0.69 mV, and −16.93 ± 0.31 mV. The changes in peak shape observed via X-ray diffraction (XRD), in vitro digestion profiles, and alterations in anthocyanin release rates under different conditions collectively indicated that the modification of ACN-NLs using SY and PPI enhanced the protective effect on the ACNs, improving their biological activity, and providing a robust foundation for the practical application of ACNs.

## 1. Introduction

Waxberry (*Myrica rubra*) is a characteristic fruit in China, celebrated for its exceptionally high anthocyanin (ACN) content [1]. ACNs are water-soluble flavonoids with demonstrated antioxidant, antibacterial, anti-inflammatory and antidiabetic properties, along with the capacity to prevent cardiovascular diseases and modulate intestinal microflora [2,3]. However, the parent nucleus and glycoside structure of ACNs contain multiple phenolic hydroxyl groups, rendering them unstable and highly sensitive to pH fluctuations, high temperatures, and light during food processing and storage. This instability often results in the loss of their biological activity [4]. Additionally, ACNs exhibit poor fat solubility, which impedes their ability to penetrate biological membranes and consequently reduces their bioavailability in humans [5]. Although ACNs can be directly absorbed in the small intestine, the complex environment of the stomach often leads to premature decomposition, further diminishing their utilization [5]. Therefore, enhancing the stability and bioavailability of ACNs has been a focal point of our research.

In recent years, nanoliposome (NL) technology has been widely applied to enhance the bioavailability of bioactive compounds. NLs are spherical vesicles primarily composed of phospholipids and cholesterol [6], which serve as carriers for bioactive substances. They protect these substances from environmental factors, thereby improving their bioavailability, targeted delivery, and sustained-release properties [7,8,9]. Despite these advantages, NLs are highly susceptible to external influences, which leads to reduced stability and utilization efficiency of the ACNs [10]. To address this issue, liposome modification with macromolecular substances has emerged as an effective strategy. This approach utilizes electrostatic interactions between macromolecules and liposomes to reduce the loss of bioactive compounds [11]. Common macromolecular modifiers include plant polysaccharides and animal proteins. Ref. [12] reported that plant polysaccharides enhanced the thermal and optical stability of astaxanthin liposomes. Tan et al. [13] utilized chitosan to modify carotenoid-loaded liposomes, resulting in improved thermal stability and dispersibility compared to conventional liposomes. Nevertheless, the application of plant proteins in nanoliposome coatings remains relatively underexplored. As a type of plant protein, pea protein isolate (PPI) exhibits advantages such as low cost, hypoallergenicity, high nutritional value, and excellent digestibility [14]. At a pH of approximately 3.0, PPI exhibits positive charges above its isoelectric point and negative charges below it [15]. By adjusting the pH, PPI can form a protective polyelectrolyte layer around negatively charged liposomes via electrostatic interactions [16]. Synanthrin (SY) is a linear anionic polysaccharide composed of fructose units linked by β-(2,1)-glycosidic bonds. Previous studies have demonstrated that the addition of SY to a Pickering emulsion of PPI has been shown to effectively immobilize proteins at the oil–water interface, enhancing the steric stability of the PPI emulsion [17]. Moreover, in combination with PPI, SY can form biopolymer complexes via a gel network structure, serving as effective carriers for the delivery of natural bioactive compounds [18]. However, the fabrication of SY-PPI-ACN-NLs via electrostatic interactions and layer-by-layer (LbL) self-assembly techniques has not yet been reported.

The surface modification of liposomes represents an effective strategy to enhance the stability and bioavailability of bioactive substances. However, limited studies have investigated the use of PPI and SY for modifying ACN-NLs. This study aimed to fabricate SY- PPI-ACN-NLs via electrostatic interactions between PPI, SY, and ACNs, thereby improving the stability of the ACNs and retarding their digestion in simulated gastric and intestinal environments. In this work, ACN-NLs were prepared using the reverse evaporation method and their formulation was optimized through orthogonal experimental design. Subsequently, the surface of the ACN-NLs was modified with PPI and SY via electrostatic adsorption. The effects of SY and PPI solution concentrations, as well as pH values, on ACN-NLs were systematically investigated, thereby establishing the formulation of an optimal preparation process for SY-PPI-ACN-NLs. The characterization of ACN-NLs and PPI-ACN-NLs was performed using X-ray diffraction (XRD) and Malvern particle size analysis. The physical and chemical properties of ACN-NLs, PPI-ACN-NLs, and SY- PPI-ACN-NLs were systematically evaluated via measurements of zeta potential, average particle size which reflects the physical stability of the systems, micromorphology and encapsulation efficiency. Furthermore, the nanoliposomes were exposed to various conditions, including different temperatures, light exposure, and simulated gastrointestinal systems. Their stability was assessed by monitoring anthocyanin release rates during a specified storage period. This research might provide valuable scientific insights into the development of SY-PPI-ACN-NLs as an efficient carrier system for bioactive substances in the food and pharmaceutical industries.

## 2. Results and Discussion

### 2.1. Optimization of Preparation Conditions for ACN-NLs via Single-Factor Experiments

#### 2.1.1. Effect of Mass Ratio of Soybean Lecithin to Cholesterol on Preparation of ACN-NLs

When the mass ratio of soybean lecithin to anhydrous ethanol was set at 3:100, with the drug-to-lipid ratio at 3:10, and the anthocyanin concentration at 3 mg/mL, the ACN-NLs were prepared by varying the mass ratio of soybean lecithin to cholesterol (1:1, 3:1, 5:1, 7:1, and 9:1). As shown in Table 1, with the increase in the mass ratio of soybean lecithin to cholesterol, the encapsulation efficiency initially showed an upward trend followed by a downward trend. The particle size demonstrated a continuous decrease, while the absolute value of the zeta potential first increased and then decreased. When the soybean lecithin-to-cholesterol mass ratio was low, phospholipid molecules dominated and were more likely to self-assemble to form a complete bilayer membrane structure. At this point, the membrane stability and the balance of the embedding space were conducive to the efficient encapsulation of ACNs. When the soybean lecithin-to-cholesterol mass ratio exceeded 5:1, the amount of soybean lecithin molecules decreased relatively, leading to disruption of the phospholipid bilayer’s structural integrity. Consequently, cholesterol could not be fully encapsulated by phospholipids and was partially exposed to the aqueous phase, disrupting membrane continuity and causing ACN leakage through membrane gaps, resulting in a decrease in encapsulation efficiency. In terms of particle size, as the ratio of soybean lecithin to cholesterol increased, the rigidity of the lipid bilayer increased while the membrane flexibility decreased. This made liposome aggregation and fusion more difficult during the preparation process, thereby causing a gradual decrease in particle size. At a 5:1 ratio, the cholesterol–lecithin balance was optimal, and the particle size was regulated by the compactness of the lipid membrane. When the ratio exceeded this threshold, the cholesterol–lecithin balance was lost, thereby resulting in an increase in particle size. When the mass ratio of soybean lecithin to cholesterol increased from 1:1 to 5:1, the absolute value of the zeta potential increased from 12.20 mV to 38.23 mV. This could be attributed to the relatively decreased proportion of cholesterol, which leads to an increased relative content of charged phospholipids within the liposomal membrane. As more charged phospholipids become exposed on the surface of the liposomes, the overall negative charge on the surface increases, resulting in a higher absolute zeta potential value. However, when the mass ratio was further increased from 5:1 to 9:1, the absolute zeta potential decreased from 38.23 mV to 32.37 mV. This phenomenon may occur because an excess of soybean lecithin causes excessive phospholipid molecules to interact with each other, leading to the encapsulation or shielding of some charged groups. Consequently, the effective surface charge is reduced, which results in a decrease in the absolute value of the zeta potential [19,20,21,22,23]. When the mass ratio of soybean lecithin to cholesterol was 5:1, the liposomes exhibited the highest encapsulation efficiency, the smallest particle size, and the highest zeta potential. Therefore, a 5:1 mass ratio of soybean lecithin to cholesterol was chosen for subsequent experiments.

#### 2.1.2. Effect of Mass Ratio of Soybean Lecithin to Anhydrous Ethanol on Preparation of ACN-NLs

When the mass ratio of soybean lecithin to cholesterol was 5:1, the drug-to-lipid ratio was 3:10, and the anthocyanin concentration was 3 mg/mL, ACN-NLs were prepared by varying the mass ratio of soybean lecithin to anhydrous ethanol to 1:100, 2:100, 3:100, 4:100, and 5:100. As shown in Table 2, with the increase in the mass ratio of soybean lecithin to anhydrous ethanol, the encapsulation efficiency first increased and then decreased. Notably, both the particle size and zeta potential remained relatively stable throughout the ratio variation, indicating the formulation’s consistency and potential for further optimization. When the mass of soybean lecithin surpassed that of cholesterol, the incomplete dissolution of soybean lecithin occurred, promoting the formation of disordered molecular aggregates. This resulted in defects in the phospholipid bilayer structure, triggering the leakage of active components (ACNs) and a decline in encapsulation efficiency. As the mass ratio of soybean lecithin to cholesterol increased, encapsulation efficiency initially rose; however, an excessively high ratio caused ethanol to over-dilute the lecithin molecules, weakening the intermolecular forces during phospholipid bilayer formation. This disrupted the membrane structure, reduced membrane integrity, and diminished encapsulation efficiency. In the emulsification–solvent evaporation method, particle size is primarily governed by process parameters (e.g., shear force and emulsification time). When the mass ratio fluctuates within a certain range, process conditions mitigate the effect of ethanol on particle size. Notably, cholesterol enhances phospholipid membrane rigidity, restricting particle aggregation and growth. The variation in the mass ratio of phospholipids to anhydrous ethanol significantly impacts particle size, its distribution, and consequently the zeta potential. Notably, the experimental data further validate this trend: particle size exhibits a discernible trend with changes in the mass ratio. Specifically, when the mass ratio reaches 5:100, the particle size increases, accompanied by a corresponding rise in the absolute value of the zeta potential. This phenomenon is underpinned by two primary mechanisms: first, the increase in particle size expands the specific surface area, altering the distribution of surface charges and thus modulating the zeta potential; second, the elevated content of soybean lecithin (as the mass ratio increases) enhances the effective particle concentration, intensifying interparticle electrostatic interactions that directly influence the zeta potential magnitude [24,25]. At a mass ratio of soybean lecithin to anhydrous ethanol of 3:100, the encapsulation efficiency was high, the particle size was relatively small, and the zeta potential was relatively high. Therefore, a mass ratio of soybean lecithin to anhydrous ethanol of 3:100 was selected for subsequent experiments.

#### 2.1.3. Influence of Drug-to-Lipid Ratio on Preparation of ACN-NLs

ACN-NLs were prepared under the conditions of a soybean lecithin-to-cholesterol mass ratio of 5:1, a soybean lecithin-to-anhydrous ethanol mass ratio of 3:100, and an anthocyanin concentration of 3 mg/mL, with drug-to-lipid ratios varying as follows: 1:10, 2:10, 3:10, 4:10, and 5:10. As shown in Table 3, with the increase in the drug-to-lipid ratio, the encapsulation efficiency initially increased and then subsequently declined. When the drug-to-lipid ratio was lower than 3:10, the saturation threshold of nanoliposome loading had not yet been reached, and the encapsulation efficiency increased with the rise in drug concentration. This is because the lipid bilayer and core voids of ACN-NLs provide limited binding sites for substance encapsulation. When the drug-to-lipid ratio exceeded this saturation threshold, the encapsulation efficiency decreased significantly. At this stage, the loading sites of the nanoliposomes are completely occupied, and excess drug molecules could not be embedded in the carrier structure. Instead, they exist in a free state or adsorb onto the carrier surface, which leads to a reduction in the measured encapsulation efficiency. When the drug concentration exceeded the saturation threshold, unencapsulated molecules tended to self-aggregate or bind to the nanocarrier surface, forming a “core-shell” or agglomerated structure. This resulted in a continuous increase in particle size, with the likelihood of high-concentration drugs disrupting the original structural integrity of ACN-NLs. With the increment in the drug-to-lipid ratio, an increasing number of ACNs are embedded into liposomes. ACNs may interact with the charges on the liposome surface, neutralizing partial negative charges and consequently leading to a decline in the absolute value of the zeta potential. Meanwhile, larger particles exhibit a relatively more dispersed surface-charge distribution, which might also contribute to the reduction in the absolute zeta potential value [26,27]. When the drug-to-lipid ratio was 3:10, the encapsulation efficiency was high, the particle size was small, and the zeta potential was high. Therefore, a drug-to-lipid ratio of 3:10 was selected for subsequent experiments.

### 2.2. Results of Orthogonal Experiment for Preparation of ACN-NLs

The orthogonal test findings are shown in Table S1. Based on the orthogonal test outcomes, the encapsulation efficiency, particle size and zeta potential were compared, where A denotes the mass ratio of soybean lecithin to cholesterol, and B signifies the mass ratio of soybean lecithin to anhydrous ethanol. C stands for drug-to-lipid ratio, revealing that the C factor had the most significant influence on ACN-NLs, followed by the B factor, with the A factor having the least influence. The optimum preparation conditions were as follows: A_2_B_2_C_2_, with a soybean lecithin-to-cholesterol ratio of 5:1, a phospholipid-to-anhydrous ethanol mass ratio of 3:100, and a drug-to-lipid ratio of 3:10. ACN-NLs obtained using these conditions exhibited the highest encapsulation efficiency, the highest absolute zeta potential, and relatively small particle size. To validate these findings, three parallel experiments were conducted according to the optimal preparation scheme, and subsequent experiments were carried out based on the orthogonal test outcomes.

### 2.3. Effects of pH and PPI Concentration on the Preparation of PPI-ACN-NLs

#### 2.3.1. Effect of pH Value on the Preparation of PPI-ACN-NLs

Based on the orthogonal optimization of ACN-NLs (with a soybean lecithin-to-cholesterol mass ratio of 5:1, soybean lecithin-to-anhydrous ethanol mass ratio of 3:100, drug-to-lipid ratio of 3:10, and anthocyanin concentration of 3 mg/mL), the PPI-ACN-NLs were prepared under different pH conditions (1, 2, 3, 4 and 5). As shown in Table 4, with the increase in pH, the encapsulation efficiency first increased and then decreased, the particle size continuously decreased and the absolute value of zeta potential first increased and then decreased. At pH 3.0, PPI reaches its isoelectric point and theoretically exhibits a net charge of zero. However, at this pH, the local positively charged regions on the PPI molecular surface exert maximum electrostatic attraction with the negatively charged groups of ACN-NLs. This interaction drives PPI molecules to tightly encapsulate ACN-NLs, thereby enhancing the encapsulation efficiency [28]. When the pH is lower than 3.0, the PPI carries a strong positive charge, whereas the negative charge density of the ACN-NLs remains relatively stable with pH changes. The increased electrostatic repulsion between the PPI molecules induces conformational extension, leading to insufficient exposure of binding sites for ACN-NLs and a subsequent decrease in encapsulation efficiency. At pH values higher than 3.0, the PPI acquires a negative charge and repels ACN-NLs due to their like charges. The weakened electrostatic attraction reduces the stability of the complex. When the pH is below 3.0, the strong positive charge of the PPI triggers intermolecular repulsion, forming a loose network structure. Encapsulating ACN-NLs under this condition generates significant steric hindrance, leading to an increase in particle size. In contrast, at the isoelectric point, the electrostatic attraction between the PPI and ACN-NLs promotes the formation of a highly ordered complex with tighter intermolecular aggregation, thereby decreasing particle size. Additionally, the low solubility of the PPI near the isoelectric point might facilitate its wrapping of ACN-NLs to exert maximum electrostatic attraction on a dense structure. When the pH exceeds 3.0, the charge repulsion between PPI and ACN-NLs hinders complex aggregation or even causes dissociation, resulting in a broader particle size distribution or particle agglomeration [29]. When the pH value rises, the concentration of OH⁻ in the solution increases, leading to the dissociation of functional groups on the particle surface. This process releases H⁺, thereby enhancing the negative charge density on the particle surface and increasing the absolute value of the zeta potential. Conversely, as the pH value decreases, the elevated H⁺ concentration neutralizes the negative charges on the particle surface, causing a reduction in the absolute value of the zeta potential [21]. Notably, PPI-modified ACNs with high encapsulation efficiency, small particle size and high zeta potential were successfully prepared at pH 3.0. Consequently, pH 3.0 was chosen for subsequent experiments.

#### 2.3.2. Effect of PPI Concentration on the Preparation of PPI-ACN-NLs

Based on the orthogonal optimization of ACN-NLs (with a soybean lecithin-to-cholesterol mass ratio of 5:1, soybean lecithin-to-anhydrous ethanol mass ratio of 3:100, drug-to-lipid ratio of 3:10 and pH adjusted to 3), varying concentrations of PPI were used (4 mg/mL, 6 mg/mL, 8 mg/mL, 10 mg/mL, and 12 mg/mL) to prepare the PPI-modified ACN-NLs. As shown in Table 5, with the increase in PPI concentration, the encapsulation efficiency first increased and then decreased, the particle size first increased and then decreased, and the absolute value of zeta potential first increased and then decreased. When the concentration of PPI was low, PPI adsorbed onto the surface of the liposomes, reduced interfacial tension, and formed a physical barrier, thereby decreasing the leakage of ACNs. When the PPI concentration exceeded the threshold (e.g., 10 mg/mL), after the liposome surface was completely covered by PPI, the additional PPI molecules remained free in the solution, inducing surface charge reversal (from negative to positive). This reversal weakened the electrostatic repulsion between liposomes, thereby promoting their aggregation and leading to a decrease in inclusion efficiency. Concomitantly, an increase in the PPI concentration induced a continuous rise in particle size, as excess PPI molecules formed bridges between liposomes, promoting aggregation via electrostatic or hydrophobic interactions. The essence of the zeta potential change lies in the charge response to the adsorption–saturation–aggregation process of PPI on the liposome surface: specifically, at low concentrations, PPI reduces the absolute value of the zeta potential through charge neutralization; importantly, at high concentrations, when surface saturation and aggregation occur, charge shielding is induced, making the zeta potential approach stability (without complete reversal) [30,31,32]. Consequently, subsequent experiments were conducted at a fixed PPI concentration set at 10 mg/mL.

### 2.4. Effect of SY Concentration on the Preparation of SY-PPI-ACN-NLs

Other conditions were kept constant, and the concentration of SY (4 mg/mL, 6 mg/mL, 8 mg/mL, 10 mg/mL, and 12 mg/mL) was varied to prepare SY-PPI-ACN-NLs. As shown in Table 6, the encapsulation efficiency first increased and then decreased with the variation in experimental parameters, while the particle size kept increasing consistently. Notably, the zeta potential remained within a stable range throughout the process, indicating the system’s colloidal stability. SY and PPI formed a complex through electrostatic interactions. PPI carries a positive charge under specific pH conditions, while SY carries a negative charge due to the dissociation of its hydroxyl groups. The two achieve layer assembly through electrostatic attraction. When the concentration of SY was low, the positive charge sites on the surface of PPI-modified liposomes were not completely occupied. With the increase in SY concentration, more SY molecules bound to the surface of PPI-modified liposomes via electrostatic interactions, forming the SY-PPI-ACN-NLs complex layer, thus contributing to a gradual improvement in encapsulation efficiency. When the SY concentration reached a certain threshold, the binding sites on the surface of PPI-modified liposomes were completely occupied, and the encapsulation efficiency reached its maximum value. If the SY concentration continued to increase, excess SY molecules could not be stably adsorbed onto the liposome surface via electrostatic interaction; instead, they form free aggregates in the system. They might even damage the integrity of the liposome membrane, causing leakage of the embedded SY and ultimately reducing the encapsulation efficiency. SY-PPI formed a multilayer structure on the liposome surface through layer-by-layer assembly: with each additional layer of the SY-PPI complex, the particle size of liposomes gradually increased due to the thickening of the shell. At low SY concentrations, the layer assembly exists as a monolayer or sparse multilayers, and the particle size increased relatively slowly. As the concentration increased, the deposition of the SY-PPI complex on the liposome surface increased, forming a thicker composite layer and leading to a continuous increase in particle size. The essence of zeta potential change reflects the interaction of surface charges between SY- and PPI-modified liposomes: at low SY concentrations, negative charges accumulate gradually with the increase in SY-binding amount, causing slight fluctuations in the absolute value of zeta potential. When the SY concentration is high, the saturation of binding sites and the effect of free SY molecules further enhance the negative charge, leading to a continuous increase in the absolute zeta potential value. However, the charge never reverses to positive, as this process is closely associated with layered assembly, charge neutralization, and the stability of liposome membranes [33,34,35]. When the SY concentration was 8 mg/mL, the encapsulation efficiency and particle size and zeta potential were 90.38%, 246.67 nm and −16.93 mV, respectively. Compared with other variables, at this concentration, the encapsulation efficiency was the highest, the particle size and the absolute zeta potential were moderate. Therefore, follow-up experiments were conducted with an SY concentration of 8 mg/mL.

### 2.5. Analysis of Appearance, Particle Size and Zeta Potential Results of ACN-NLs, PPI-ACN-NLs and SY-PPI-ACN-NLs

Encapsulation efficiency, particle size, and zeta potential are crucial parameters for characterizing liposomes, reflecting their physical properties, stability, and uniformity [36]. From Figure S1 and as shown in the color differences, the color difference measurements for ACN-NLs, PPI-ACN-NLs, and SY-PPI-ACN-NLs are 36.79, 34.74, and 30.52, respectively. This corresponds to the appearance of deep purple, light purple, and light pink, with no precipitates or flocculation, and they exhibited uniform distributions. As can be seen from Table 7, the encapsulation efficiencies of ACN-NLs, PPI-ACN-NLs, and SY-PPI-ACN-NLs were 52.59%, 83.80%, and 90.38%, respectively, the averages particle sizes were 134.60 nm, 213.20 nm, and 246.60 nm, respectively, and the zeta potentials were −32.40 mV, −27.46 mV, and −16.93 mV, respectively. According to the above data, ACN-NLs were successfully prepared, but still exhibited low encapsulation efficiency. Studies have shown that the presence of lipids, polysaccharides, and proteins in the biological membranes can help enhance its stability and function in the liposome model [37]. The encapsulation efficiency and particle size of PPI-ACN-NLs formed after PPI modification were both increased, and the absolute value of zeta potential was reduced. This might be attributed to the adsorption of the positively charged PPI solution and negatively charged ACN-NLs onto the surface of liposomes through electrostatic interaction, thereby closely bonding them to improve the encapsulation efficiency [38]. At the same time, the addition of PPI led to an increase in particle size, and the absolute value of the liposome zeta potential decreased after the electrostatic interaction of positive and negative charges. All of the above indicate that PPI successfully modified ACN-NLs. SY-PPI-ACN-NLs were formed after PPI-ACN-NL modification, and the encapsulation efficiency and particle size both increased, while the absolute value of the zeta potential decreased. This might be because proteins and polysaccharides were adsorbed onto the surface of the liposomes through electrostatic interaction and layer-by-layer self-assembly to improve their stability. This indicates that the nanoparticle distribution of liposomes was relatively uniform, the system was relatively stable, and the static flocculation phenomenon does not occur easily [39]. The absolute value of the zeta potential was 16.93 mV, indicating that the repulsion between charged particles was large, and the liposomes were not prone to aggregation during storage [40]. All the above indicate that ACN-NLs, PPI-ACN-NLs, and SY-PPI-ACN-NLs were successfully prepared.

### 2.6. ABTS^+^ Free Radical Scavenging Capacity of ACN-NLs, PPI-ACN-NLs and SY-PPI-ACN-NLs

**Figure 1:**
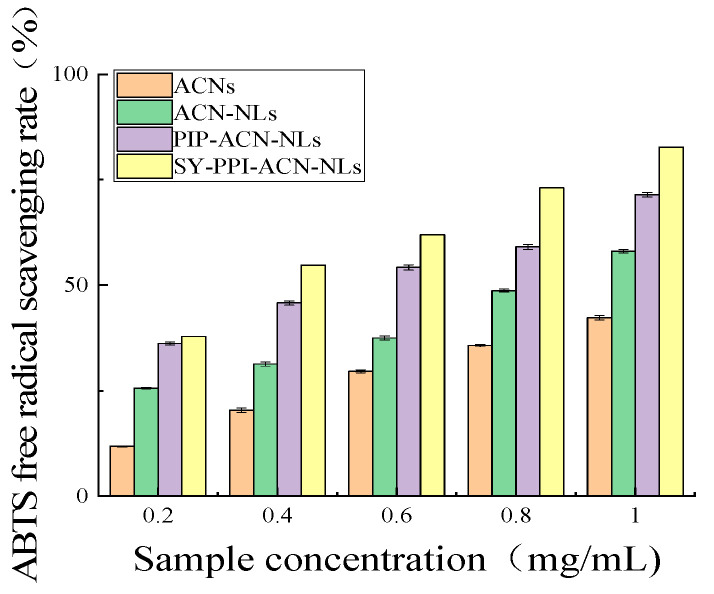
ABTS^+^ free-radical-scavenging capacity of ACNs, ACN-NLs, PPI-ACN-NLs and SY-PPI-ACN-NLs. Note: anthocyanins (ACNs), ACN-NLs (anthocyanin nanoliposomes), PPI-ACN-NLs (pea protein isolate-modified anthocyanin nanoliposomes), SY-PPI-ACN-NLs (synanthrin, pea protein isolate-modified anthocyanin nanoliposomes).

As shown in Figure 1, with the increase in the concentrations of ACNs, ACN-NLs, PPI-ACN-NLs and SY-PPI-ACN-NLs, the scavenging rate of ABTS^+^ radicals was enhanced. At a concentration of 0.2 mg/mL, the ABTS^+^ radical scavenging rates of ACNs, ACN-NLs, PPI-ACN-NLs, and SY-PPI-ACN-NLs were 11.80%, 25.65%, 36.16%, and 37.85%, respectively; when the concentration was 0.4 mg/mL, those rates for ACNs, ACN-NLs, PPI-ACN-NLs and SY-PPI-ACN-NLs were 20.40%, 31.30%, 45.78% and 54.75%, respectively; when the concentration was 0.6 mg/mL, the corresponding values of ACNs, ACN-NLs, PPI-ACN-NLs and SY-PPI-ACN-NLs were 29.60%, 37.50%, 54.19% and 61.95%, respectively; when the concentration was 0.8 mg/mL, the values of ACNs, ACN-NLs, PPI-ACN-NLs and SY-PPI-ACN-NLs were 35.70%, 48.71%, 59.06% and 73.09%, respectively; when the concentration was 1.0 mg/mL, those of ACNs, ACN-NLs, PPI-ACN-NLs and SY-PPI-ACN-NLs were 42.30%, 58.05%, 71.47% and 82.74%, respectively. At these concentrations, the scavenging capacity order followed SY-PPI-ACN-NLs > PPI-ACN-NLs > ACN-NLs > ACNs. Notably, the scavenging capacity of ACN-NLs was significantly superior to that of free ACNs, confirming that nanoliposomes play a protective role in the antioxidant activity of ACNs. This is attributed to the bilayer phospholipid structure of liposomes providing a physical barrier for ACNs, effectively reducing their direct contact with the oxidative environment and decreasing the degradation of active components [41]. Furthermore, the scavenging capacity of PPI-ACN-NLs was further improved, indicating that the modification of polyelectrolyte PPI might optimize the antioxidant performance of ACNs by enhancing the capacity of liposomes or changing their interaction mode with free radicals. It is worth noting that SY-PPI-ACN-NLs exhibited the best free radical scavenging capacity. This synergistic effect might originate from the fact that SY (as a natural polyphenol, which itself has antioxidant activity and might improve the surface charge, hydrophilic–lipophilic balance, or membrane fluidity of liposomes) modification endows liposomes with excellent interfacial properties, enhancing the encapsulation capacity and controlled-release capacity of the carrier for ACNs, and thus improving the overall antioxidant effect [42].

### 2.7. X-Ray Diffraction (XRD) Analysis by ACN-NLs, PPI-ACN-NLs and SY-PPI-ACN-NLs

**Figure 2:**
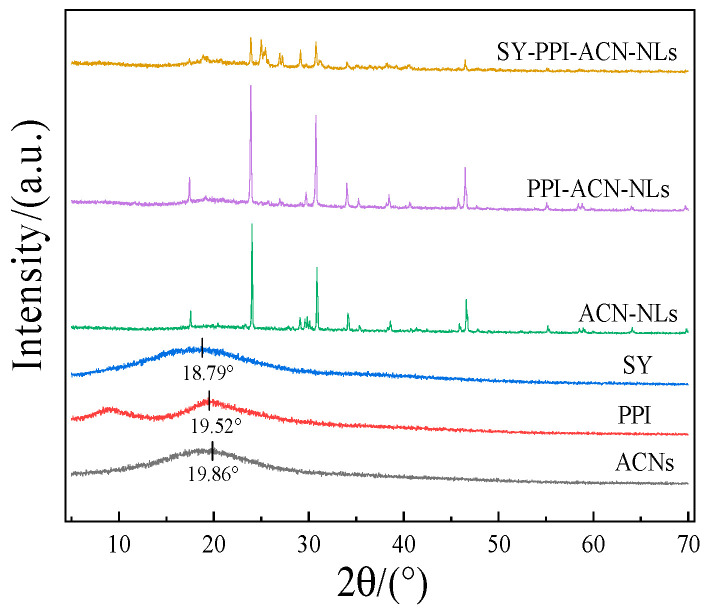
X-ray diffraction patterns of ACNs, PPI, SY, ACN-NLs, PPI-ACN-NLs and SY-PPI-ACN-NLs. Note: anthocyanins (ACNs), ACN-NLs (anthocyanin nanoliposomes), PPI-ACN-NLs (pea protein isolate-modified anthocyanin nanoliposomes), SY-PPI-ACN-NLs (synanthrin, pea protein isolate-modified anthocyanin nanoliposomes).

X-ray diffraction (XRD) is an effective method by which to evaluate the complexation between substances. Crystallinity can be reflected by the peak shape in the X-ray diffraction pattern. This allows observation of liposome formation by ACNs, as well as changes in the crystal morphology of liposomes after PPI and SY modification, to understand substance interactions. A sharp and narrow diffraction peak indicates high crystallinity, whereas a broad peak suggests an amorphous state [43]. In this study, the XRD patterns of ACNs, PPI, SY, ACN-NLs, PPI-ACN-NLs and SY-PPI-ACN-NLs were observed over a 2*θ* range of 5–70°. The results for SY are shown in Figure 2 where ACNs, PPI and SY exhibit broadened diffraction peaks at 2*θ* of 19.86°, 19.52° and 18.79°. These diffuse features indicate an amorphous state, consistent with the absence of sharp crystalline reflections, which suggests that all samples exist in an amorphous state with an amorphous structure. ACN-NLs, PPI-ACN-NLs and SY-PPI-ACN-NLs showed multiple separated and sharp diffraction peaks, indicating a high degree of crystallinity, without the characteristic peaks of ACN, PPI and SY, which might be attributed to the interaction between the ACNs and phospholipid molecules during the preparation of liposomes. The original structure was changed, resulting in enhanced crystallinity [44], indicating that ACNs were successfully embedded by liposomes. At the same time, PPI and SY also modified the liposome surface structures through electrostatic interaction and layer-by-layer self-assembly adsorption. It can be seen that PPI and SY successfully modified ACN-NLs to form PPI-ACN-NLs and SY-PPI-ACN-NLs, which changed the particle size, properties and microstructure of the liposomes.

### 2.8. In Vitro Digestive Analysis of ACN-NLs, PPI-ACN-NLs and SY-PPI-ACN-NLs

**Figure 3:**
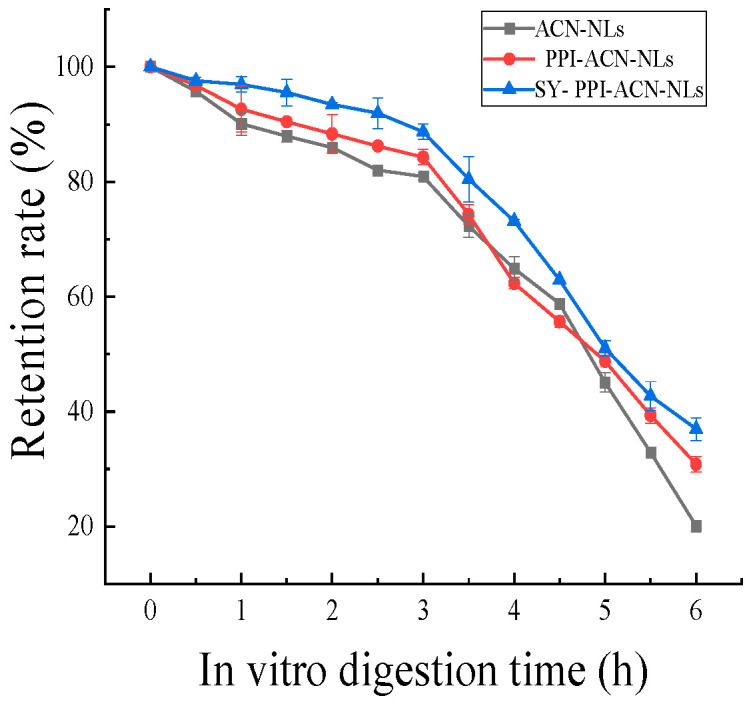
Simulated retention rates of ACN-NLs, PPI-ACN-NLs and SY-PPI-ACN-NLs. Note: ACN-NLs (anthocyanin nanoliposomes), PPI-ACN-NLs (pea protein isolate-modified anthocyanin nanoliposomes), SY-PPI-ACN-NLs (synanthrin, pea protein isolate-modified anthocyanin nanoliposomes).

Studies have demonstrated that an increased retention rate during in vitro digestion is conducive to nutrient absorption [45]. As illustrated in Figure 3, the retention rates of ACN-NLs, PPI-ACN-NLs, and SY-PPI-ACN-NLs were evaluated across SS, SGF, and SIF stages. At the 0–1 h mark, the samples entered the SS stage with retention rates recorded at 90.07 ± 0.71%, 92.63 ± 0.49%, and 96.95 ± 1.20% for ACN-NLs, PPI-ACN-NLs, and SY-PPI-ACN-NLs, respectively. Between 1 and 3 h, as the samples transitioned into the SGF stage, their respective retention rates were observed to be 80.93 ± 0.38%, 84.29 ± 0.74%, and 88.69 ± 0.30%. Following this period, from 3 to 6 h when entering the SIF stage, the retention rates decreased significantly to 20.10 ± 0.58%, 30.83 ± 0.42%, and 36.97 ± 0.26% for ACN-NLs, PPI-ACN-NLs, and SY-PPI-ACN-NLs, respectively. It is evident that ACN-NLs exhibit relative stability during the SGF stage; likely this might be attributed to a pH value of approximately 2 in this environment where anthocyanins tend to remain stable under strongly acidic conditions. Conversely, during both SS and SIF stages characterized by a neutral pH of around 6.8, the anthocyanins demonstrate instability leading to rapid declines in their retention rates [46]. Throughout the in vitro digestion processes, it was noted that the retention rate of PPI-ACN-NLs surpassed that of standard ACN-NLs; this can be explained by how the liposomal phospholipid bilayers effectively shield the ACN-NLs from external environmental interference while simultaneously reducing their oxidation and decomposition rates—thereby enhancing their overall retention [47]. The retention rate of SY-PPI-ACN-NLs is higher than that of PPI-ACN-NLs. This is because SY-PPI-ACN-NLs, formed by the combination of PPI and ACN-NLs through electrostatic interaction, can further reduce the degradation of ACN-NLs in the digestion process, thereby improving retention. The bioavailability of ACN-NLs, PPI-ACN-NLs, and SY-PPI-ACN-NLs was 20.10 ± 0.58%, 30.83 ± 0.42%, and 36.97 ± 0.26%, respectively.

### 2.9. Analysis of Stability of ACN-NLs, PPI-ACN-NLs and SY-PPI-ACN-NLs at Different Temperatures

The three samples of ACN-NLs, PPI-ACN-NLs, and SY-PPI-ACN-NLs were stored at 5 °C, 25 °C, 45 °C, and 65 °C for 30 days. Changes in their appearance, ACN release rate, and MDA values were observed and are depicted in Figures S2 and S3. According to their appearance in Figure S2, there was no significant change in the appearance of the samples stored at 5 °C. During storage at 25 °C, the color of the samples slightly lightened without stratification. At 45 °C, the color of ACN-NLs darkened with less obvious stratification; PPI-ACN-NLs and SY-PPI-ACN-NLs changed from light pink to light green, also with less obvious stratification. However, at 65 °C, the color of ACN-NLs darkened significantly with obvious stratification, and PPI-ACN-NLs and SY-PPI-ACN-NLs also became darker with clear stratification. It is evident that PPI-ACN-NLs and SY-PPI-ACN-NLs are more stable than ACN-NLs at 5 °C.

Observing the changes in ACN release rates and MDA values in Figure S3, these results indicate that at 5 °C, the release rates and MDA values of ACN-NLs, PPI-ACN-NLs, and SY-PPI-ACN-NLs were 15.65%, 13.47%, 10.30%, and 2.27 µg/mL, 2.17 µg/mL, and 1.90 µg/mL, respectively. At 25 °C, the release rates and MDA values of ACN-NLs, PPI-ACN-NLs, and SY-PPI-ACN-NLs were 26.3%, 21.8%, and 17.3%, respectively, with corresponding MDA values of 2.48 µg/mL, 2.25 µg/mL, and 2.16 µg/mL. At 45 °C, the release rates and MDA values were 44.7%, 37.5%, and 31.4%, respectively, with MDA values of 2.65 µg/mL, 2.33 µg/mL, and 2.17 µg/mL. At 65 °C, the release rates and MDA values of ACN-NLs, PPI-ACN-NLs, and SY-PPI-ACN-NLs were 62.7%, 43.8%, 39.4%, and 2.78 µg/mL, 2.39 µg/mL, and 2.29 µg/mL, respectively. From these results, it is clear that with increasing storage time and temperature, the ACN release rate of the samples continues to increase. The growth rate is faster in the first 10 days and then becomes relatively gentle. The release rate follows the order of SY-PPI-ACN-NLs < PPI-ACN-NLs < ACN-NLs, which might be attributed to the gradual increase in the release rate of the samples with temperature. This intensifies the hydrolysis and oxidation of phospholipids, resulting in the destruction of the lipid bilayer and the leakage of ACNs from the liposomes [48]. SY and PPI-modified liposomes form a protective layer on the surface of the liposomes, which can reduce ACN leakage. Therefore, the stability of SY-PPI-ACN-NLs is higher than that of PPI-ACN-NLs and ACN-NLs. With the increase in storage time and temperature, the MDA values of the sample kept increasing. The growth rate was faster in the first 5 days and then became relatively mild, following SY-PPI-ACN-NLs < PPI-ACN-NLs <ACN-NLs, because the fatty acid chain of phospholipids is oxidized during the storage of liposomes after preparation. Liposomes modified with SY and PPI can protect phospholipids and reduce the oxidation rate, thus improving their stability [49].

As shown in Table 8, the half-lives of ACN-NLs, PPI-ACN-NLs and SY-PPI-ACN-NLs increased gradually in sequence at different temperatures, and the release of ACNs was better described by first-order degradation kinetics. Therefore, a storage temperature of 5 °C was more suitable for maintaining the stability of the liposomes.

### 2.10. Analysis of Storage ACN-NLs, PPI-ACN-NLs and SY-PPI-ACN-NLs of Liposomes Under Light

The three samples ACN-NLs, PPI-ACN-NLs, and SY-PPI-ACN-NLs were stored under light and dark conditions for 30 days, and changes in their appearance, ACN release rates, and MDA values were observed, as shown in Figures S4 and S5. According to the images in Figure S4, during the light storage period, the color of the samples gradually became lighter, and the degree of green in the samples showed ACN-NLs > PPI-ACN-NLs > SY-PPI-ACN-NLs without stratification. During the dark storage period, the color of the samples deepened slightly, and there was no stratification. It can be seen that it is beneficial for liposome storage to avoid storage in the dark.

By observing the changes in ACN release rate and MDA values in Figure S5, these results indicate that under light storage, the release rates and MDA values of ACN-NLs, PPI-ACN-NLs, and SY-PPI-ACN-NLs were 37.30%, 28.40%, 23.80%, and 2.48 μg/mL, 2.25 μg/mL, 2.16 μg/mL, respectively. The release rates and MDA values of ACN-NLs, PPI-ACN-NLs, and SY-PPI-ACN-NLs stored under dark conditions were 26.30%, 21.80%, and 17.30%, respectively, and 2.22 μg/mL, 1.93 μg/mL, 1.85 μg/mL; it can be seen from the above results that with the increase in storage time, the ACN release rates of the samples continue to increase, and the gentle increase is faster initially, within the first 5 days, and then it becomes relatively more gentle, and the release rate is in the order of SY-PPI-ACN-NLs < PPI-ACN-NLs < ACN-NLs. This might be because light will accelerate the decomposition of liposomes and lead to the leakage of anthocyanins easily. However, liposomes modified by SY and PPI can be closely bonded with liposomes through electrostatic action to form a protective film, which can reduce the leakage of ACNs. Therefore, the stability of SY-PPI-ACN-NLs is higher than that of PPI-ACN-NLs and ACN-NLs. With the increase in storage time and temperature, the MDA values of the samples keep increasing. The gentle increase was faster in the first 5 days and then became relatively gentler, following SY-PPI-ACN-NLs < PPI-ACN-NLs <ACN-NLs, because the fatty acid chain of phospholipids will be oxidized during the storage of liposomes after preparation. Liposomes modified by SY and PPI can protect phospholipids and reduce the oxidation rate, thus improving their stability [50].

As shown in Table 9, the half-lives of ACN-NLs, PPI-ACN-NLs and SY-PPI-ACN-NLs increased under both light and dark conditions, and the release of ACNs was better described by first-order degradation kinetics. Therefore, the storage environment away from light was more suitable for the preservation of these liposomes.

### 2.11. Analysis of Storage Stability Results of ACN-NLs, PPI-ACN-NLs and SY-PPI-ACN-NLs in Beverage Model

The ACN-NLs, PPI-ACN-NLs and SY-PPI-ACN-NLs samples were stored in four food simulants (hydrophilic substances (10% ethanol), alcoholic substances (20% ethanol), lipophilic substances (50% ethanol), and acidic substances (3% acetic acid)) for 30 days. Changes in appearance, ACN release rates, and MDA values were monitored, results are presented in Figures S6 and S7. According to the visual observations depicted in Figure S6, ACN-NLs exhibited no significant changes across all four food simulants. In contrast, PPI-ACN-NLs showed stratification after 10 days of storage in 3% acetic acid, while no discoloration or precipitation occurred in the other three simulants during storage. For SY-PPI-ACN-NLs, stratification was observed after 10 days of storage in 20% ethanol, 50% ethanol, and 3% acetic acid; however, no color change or precipitation occurred during storage in hydrophilic substances. These findings suggest that 10% ethanol is more conducive to liposome stability.

By analyzing the changes in ACN release rates and MDA values, as shown in Figure S7, it was observed that during storage in 10% ethanol, the release rates of ACN-NLs, PPI-ACN-NLs, and SY-PPI-ACN-NLs were 38.90%, 23.20%, and 14.37%, respectively, while the corresponding MDA values were 1.81 µg/mL, 1.61 µg/mL, and 1.54 µg/mL. In 20% ethanol, these values were 39.40%, 23.40%, 14.18% (release rates) and 1.84 µg/mL, 1.66 µg/mL, 1.55 µg/mL (MDA). In 50% ethanol, the release rates increased to 45.86%, 30.61%, 18.37%, and MDA values reached 2.21 µg/mL, 2.04 µg/mL, 1.89 µg/mL. In 3% acetic acid, the highest release rates (47.66%, 37.31%, 26.56%) and MDA values (2.51 µg/mL, 1.90 µg/mL, 1.58 µg/mL) were observed.

This suggests that PPI and SY modifications reduce ACN release, zeta potential attributed to electrostatic interactions and layer-by-layer self-assembly between PPI/SY and negatively charged ACN-NLs, which enhances the protection of ACN biological activity [51]. Among the simulants, ACN-NLs, PPI-ACN-NLs, and SY-PPI-ACN-NLs showed higher release rates in 50% ethanol than in 20% or 10% ethanol, likely because increased ethanol concentration promotes phospholipid decomposition in liposomes, enhancing bioactive substance permeability [52]. Additionally, 3% acetic acid induced the highest release rates, possibly attributable to the acidic conditions destabilizing the NLs and facilitating ACN release [53]. Notably, SY/PPI-modified liposomes exhibited reduced sensitivity to acidic conditions, leading to lower ACN release. These results indicate that the ACN release rate increased with storage time in the order given. As shown in Figure S7, MDA levels in all formulations increased over time across the four food simulants. The MDA levels increased rapidly during the first 15 days and then increased more gradually, indicating that lipid oxidation rates initially accelerated before slowing. Significantly, PPI-ACN-NLs and SY-PPI-ACN-NLs showed lower MDA levels than ACN-NLs, confirming that PPI and SY modifications mitigate lipid oxidation.

As shown in Table 10, the half-lives of ACN-NLs, PPI-ACN-NLs and SY-PPI-ACN-NLs progressively increased across the four simulants, with ACN-NLs exhibiting behavior consistent with first-order degradation kinetics. These findings underscore the potential application of zeta potential of ACN-NLs, PPI-ACN-NLs and SY-PPI-ACN-NLs for food products.

## 3. Materials and Methods

### 3.1. Materials

Waxberry anthocyanins (25%) were supplied by Shaanxi Meixner Biotechnology Co., Ltd. (Xian, China). Tween 80 was purchased from Shanghai Ben Chemical Co., Ltd. (Shanghai, China). Soybean lecithin, cholesterol, and anhydrous ethanol were obtained from Maclean (Shanghai, China) and Xiong Technology (Shantou, China), respectively. Pea protein isolate (>80%), pepsin (activity: 3000–3500 NF), trypsin (>3000 units/g), and bile salts were provided by Yanaye Biotechnology Co., Ltd. (Shanghai, China). Analytical-grade reagents including potassium dihydrogen phosphate (K_2_HPO_4_, >90%), glacial acetic acid (CH_3_COOH, >90%), concentrated hydrochloric acid (HCl, >90%), sodium hydroxide (NaOH, >90%), 2,2′-Azinobis-(3-ethylbenzthiazoline-6-sulphonate) (ABTS, >90%), potassium persulfate (K_2_S_2_O_8,_ >90%), and others were purchased from the China National Pharmaceutical Group Corporation (Beijing, China).

### 3.2. Methods

#### 3.2.1. Production of ACN-NLs

ACN-NLs were prepared by reverse evaporation method [54]. Soybean lecithin mix was obtained from soybean lecithin and cholesterol at a 5:1 ratio (*w*/*w*), then the mixture was dissolved in 30 mL of anhydrous ethanol under magnetic stirring until a clear, homogeneous solution was obtained. A sample of 4 mg/mL anthocyanin (ACN) phosphate buffered saline (PBS) solution (0.2 M, pH 3.5) was prepared as the aqueous phase. The organic phase was injected into the water phase, hydrated with Tween 80 at 45 °C for 30 min, then treated with ultrasonic treatment (Shanghai Jingqi Instrument Co., Ltd., Model: UC-300VDE, Power 300W, Shanghai, China) in an ice water bath for 2 min, and then concentrated under a rotary evaporator (Shanghai Yarong, model: RE2000 series, Shanghai, China) at 45 °C to completely remove the organic solvent. The concentrated nanoliposome solution was filtered through a 0.22 μm filter membrane to obtain ACN-NLs.

#### 3.2.2. Process Optimization of ACN-NLs via Reverse Evaporation Method

According to the preparation method described in 3.2.1, single-factor trials were conducted to investigate the mass ratios of soybean lecithin to cholesterol (1:1, 3:1, 5:1, 7:1, and 9:1), phospholipids to anhydrous ethanol (1:100, 2:100, 3:100, 4:100, and 5:100), and drugs to lipids (1:10, 2:10, 3:10, 4:10, and 5:10).

#### 3.2.3. Orthogonal Test of ACN-NLs

According to the results of the single factor experiment, the orthogonal test table of three factors and three levels was designed. The three factors were the mass ratio of soybean lecithin to cholesterol (1:1, 5:1 and 9:1), the mass ratio of phospholipid to anhydrous alcohol (2:100, 3:100 and 4:100), and the ratio of drug to lipid (1:10, 2:10, and 3:10), respectively. The encapsulation efficiency and particle size were used as indexes to obtain the best preparation conditions for liposomes.

#### 3.2.4. Preparation of PPI and SY Reserve Solution

The appropriate amount of PPI solution and SY solution were prepared at the concentration of 20%, *w*/*v*. The PPI and SY solution were stirred with a magnetic stirrer until completely dissolved, and then treated with an ultrasonic water bath for 15 min (40 kHz, 25 °C). After ultrasonic treatment, the solution was stored in a refrigerator at 4 °C for future use.

#### 3.2.5. Preparation of Anthocyanin Nanoliposomes Modified by PPI

According to Liu et al.’s method [55], PPI-ACN-NLs were prepared by appropriate modification. PPI reserve solution (20%, *w*/*v*) was diluted with acetate buffer solution (0.05 M, pH 1, 2, 3, 4, and 5) to prepare different concentrations of PPI (4%, 6%, 8%, 10% and 12%, *w*/*v*). Then ACN-NL droplets were added to the PPI solution at a ratio of 1:1. It was stirred continuously on a magnetic stirrer for 2 h to form PPI-CAN-NLs, and the PPI-ACN-NLs produced were stored at 4 °C for later use. Encapsulation efficiency particle size and zeta potential, PDI were measured to find the optimal pH value and PPI concentration.

#### 3.2.6. Preparation of Bilayer Modified Anthocyanin Nanoliposomes with SY and PPI

According to the method of Liu et al. [56], SY-PPI-ACN-NLs were prepared with appropriate modification. Different SY solutions (4%, 6%, 8%, 10%, and 12%, *w*/*v*) were prepared by diluting the SY stock solution (20%, *w*/*v*) with acetate buffer solution (0.05 M, pH 3). The PPI-ACN-NLs were added to the SY solution at a ratio of 1:1. The mixture was continuously stirred on a magnetic stirrer for 2 h to form SY-PPI-ACN-NLs, which were stored at 4 °C for subsequent use. The encapsulation efficiency and particle size were measured to determine the optimal SY concentration.

### 3.3. Characterization of ACN-NLs, PPI-ACN-NLs and SY-PPI-ACN-NLs

#### 3.3.1. Encapsulation Efficiency and Release Rate of ACN-NLs, PPI-ACN-NLs, and SY-PPI-ACN-NLs

Anthocyanin powder was prepared as a 1 mg/mL stock solution, which was then diluted into 0.2 mg/mL, 0.4 mg/mL, 0.6 mg/mL, 0.8 mg/mL, and 1 mg/mL The absorbance was detected at a wavelength of 517 nm. Linear regression analysis of ACN mass concentration (X) and absorption value (Y) yield, using the equation: y = 0.354x − 0.002 (R^2^ = 0.998). This equation has a good linear relationship when the mass concentration of ACN is 0.00–1.00 mg/mL. Centrifugal–ultraviolet spectrophotometry was used to determine the encapsulation efficiency of ACN-NLs, and the specific operation was slightly modified by referring to the method of Gorja et al. [57]. A measure of 0.5 mL of ACN-NLs was drawn into a volumetric flask and ultrapure water was added to adjust the volume to the calibration mark. Then, the aqueous solution of ACN-NLs was centrifuged at 8000 rpm for 30 min. The supernatant was taken and its absorbance value was detected at 517 nm. The concentration of free ACNs was calculated by using the regression equation. An accurate measure of 0.5 mL of ACN-NLs was taken and transferred to a 10 mL volumetric flask. Subsequently, 4.5 mL of anhydrous ethanol was added, followed by the addition of ultrapure water to adjust the final volume to the mark, and it was then immediately transferred to a 50 mL centrifuge tube at 8000 rpm for 30 min. The supernatant was taken, the absorbance value was measured, and the concentration of free ACN (C_2_) was calculated by inserting it into the linear regression equation. The encapsulation efficiency and release rate are calculated as follows:

(1) $$EE\left(\%\right)=\left(1-\frac{C_{1}}{C_{2}}\right)\times 100\%$$

(2) $$Release\left(\%\right)=\frac{C_{1}}{C_{2}}\times 100\%$$

C_1_ is the total drug added to the sample (ACNs), mg; C_2_ is the free non-embedded drug (ACNs), mg.

#### 3.3.2. Determination of Particle Size and Zeta Potential for ACN-NLs, PPI-ACN-NLs, and SY-PPI-ACN-NLs

The appropriate amount of ACN-NLs, PPI-modified ACN-NLs and SY-PPI-modified ACN-NLs was diluted 25 times with ultrapure water (Heal Force, Lixin Instrument Co., Ltd., Shanghai, China). The average particle size and zeta potential of the liposomes were determined by Malvern particle size meter (Mastersizer 3000, Malvern PANalytical, Worcestershire, Britain). The samples were equilibrated at 25 °C for 120 s, and each measurement was repeated 3 times [58].

#### 3.3.3. Determination of Color Difference for ACN-NLs, PPI-ACN-NLs and SY-PPI-ACN-NLs

The apparent colors of ACN-NLs, PPI-modified ACN-NLs and SY-PPI-modified ACN-NLs were determined using a CR-10 colorimeter. (Konica Minolta, Japan). The values of L, a and b were determined. The color difference was calculated as follows [59].

(3) $$∆E=\sqrt{{\left(L_{1}-L_{0}\right)}^{2}+{\left(a_{1}-a_{0}\right)}^{2}+{\left(b_{1}-b_{0}\right)}^{2}}$$

where ΔE represents the color difference value; L_1_, a_1_ and b_1_ are the chromaticity values of liposomes; L_0_, a_0_ and b_0_ are the chromaticity values of liposomes in the control group.

#### 3.3.4. Determination of ABTS Free Radical Scavenging Efficiency in ACN-NLs, PPI-ACN-NLs and SY-PPI-ACN-NLs

The ABTS free radical scavenging efficiency of liposomes was assessed using a modified method described by Herath et al. [53]. Preparation of ABTS working solution: 7 mmol/L ABTS solution was mixed with 2.45 mmol/L potassium persulfate solution in equal volumes, and left at room temperature in the dark overnight (12–16 h) to prepare the ABTS^+^ free radical stock solution for future use. The stock solution was diluted with anhydrous ethanol to adjust its absorbance to 0.7 ± 0.02 at 734 nm, forming the ABTS working solution. Specifically, 0.5 mL of the sample solution was mixed with 5.0 mL of ABTS solution and incubated in the dark for 30 min. The absorbance was subsequently measured at 734 nm and recorded as A_1_. For the blank control, 0.5 mL of anhydrous ethanol was mixed with 5.0 mL of ABTS solution under the same conditions, and the absorbance was measured at 734 nm, denoted as A_0_. Each experimental group was repeated three times to ensure reliability. The formula for calculating the ABTS free radical scavenging efficiency is as follows:

(4) $$ABTS=\left(1-\frac{A_{0}}{A_{1}}\right)\times 100\%$$

where ABTS represents the free radical scavenging efficiency (%); A_0_ denotes the absorbance of the blank control group; A_1_ corresponds to the absorbance of the sample.

#### 3.3.5. X-Ray Diffraction (XRD) Analysis of ACN-NLs, PPI-ACN-NLs, and SY-PPI-ACN-NLs

The crystal structures of ACN-NLs, PPI-modified ACN-NLs and SY-PPI-modified ACN-NLs were determined using an X-ray Cu-Ka diffractometer (Brook Company, Solingen, Germany). The scattering angle (2θ) was measured from 5° to 70° at a scan speed of 10 degrees per minute [60]

#### 3.3.6. Determination of Malondialdehyde (MDA) Content

The lipid oxidation degree of ACN-NLs, PPI-ACN-NLs, and SY-PPI-ACN-NLs was calculated using the TBA (thiobarbituric acid) reaction method. A certain amount of ACN-NLs, PPI-ACN-NLs, and SY-PPI-ACN-NLs was weighed and then mixed with the corresponding amount of 5% trichloroacetic acid. First, 5mL of the solution was drawn from the mixed solution and mixed with an equal volume of TBA solution at a concentration of 0.02 mol/L. The newly prepared mixture was heated in a constant-temperature water bath at 80 °C for color development, and then gradually cooled to room temperature. The absorbance of the sample at 535 nm was measured using a visible spectrophotometer, and the MDA value was calculated accordingly. ACN-NLs, PPI-ACN-NLs, and SY-PPI-ACN-NLs were removed at 0, 5, 10, 15, 20, 25, and 30 days, and the experiment was conducted according to [61]. The MDA content was calculated using the following formula.

(5) $$MDA(µg/mL)=A_{535}nm\times 0.83$$

### 3.4. In Vitro Digestion Study of ACN-NLs, PPI-ACN-NLs and SY-PPI-ACN-NLs

The digestive fluids were prepared following the method described by Urszula [62]. Simulated saliva (SS) was obtained by dissolving 2.38 g of Na_2_HPO_4_, 0.19 g of KH_2_PO_4_, and 8 g NaCl in 1000 mL of deionized water at a pH of 6.8, with the addition of 200 U of α-amylase. Simulated gastric fluid (SGF) was prepared by dissolving 2.0 g NaCl, 7 mL of concentrated HCl, and 3.2 g of pepsin in 1000 mL of deionized water; Simulated intestinal fluid (SIF) was prepared by dissolving 3.4 g KH_2_PO_4_ in 1000 mL of deionized water, adjusting the pH to 6.8 using 0.1 mol/L of NaOH, and adding 5 g of pancreatic enzyme to 500 mL of deionized water.

The measured 7.5 mL samples were mixed with an equal volume of simulated saliva (SS), and the pH was adjusted to 6.8. The samples were digested in a constant temperature water bath at 37 °C (100 rpm/min) for 1 h. Following oral digestion, 7.5 mL of digestive fluid was taken and mixed with an equal volume of simulated gastric fluid (SGF), with the pH adjusted to 2.0, The digestive fluid was incubated at 37 °C in a water bath shaker (100 rpm/min) for 2 h. After gastric digestion, 7.5 mL of digestive fluid was taken, and mixed with an equal volume of simulated intestinal fluid (SIF), with the pH was adjusted to 6.8. The digestion was conducted at 37 °C in a water bath shaker (100 rpm/min) for 3 h. The sample was easily degraded after digestion, and the whole digestion process was maintained at 37 °C, resulting in sample loss after simulated in vitro digestion. The retention rate of the sample after in vitro digestion is calculated as shown in Formula (6). The bioavailability of the samples after digestion was determined using the method described by Zhang et al. [63]; and the calculation of bioavailability is shown in Formula (7):

(6) $$Retentionrate\left(\%\right)=\frac{W_{1}}{W_{2}}\times 100\%$$

where W_1_ is the anthocyanin content (mg) in the digestive fluid; W_2_ is the initial anthocyanin content (mg) in the sample.

(7) $$bioavailability\left(\%\right)=\frac{C_{2}}{C_{1}}\times 100\%$$

where C_2_ is the anthocyanin content (mg) in the sample after digestion; C_1_ is the initial anthocyanin content (mg) in the sample.

### 3.5. Study of Storage Stability of ACN-NLs, PPI-ACN-NLs and SY-PPI-ACN-NLs

#### 3.5.1. Stability Experiment of ACN-NLs, PPI-ACN-NLs and SY-PPI-ACN-NLs at Different Temperatures

The stability of ACN-NLs, PPI-ACN-NLs and SY-PPI- ACN-NLs at various temperatures was investigated. Twenty milliliters of each, ACN-NLs, PPI-ACN-NLs and SY-PPI-ACN-NLs, was respectively placed in five glass bottles and stored at different temperatures (5 °C, 25 °C, 45 °C, 65 °C) in the dark for 30 days, with samples taken every five days [57]. Observation and characterization were conducted, and the anthocyanin release rate was assayed according to Section 3.3.1, while MDA was determined according to Section 3.3.6. Furthermore, zero-order and first-order degradation kinetics of the ACNs in the food simulation system were simulated, with specific release kinetics equations as presented in Formulas (8)–(10).

(8) Zero order kinetic equation: Q=K×t

(9) $$\text{First-order}\;\mathrm{kinetic}\;\mathrm{equation}:\;\ln\left(1-Q\right)=-Kt$$

(10) Half-life period: t12=In2K

In the Formula, Q represents the total release amount, K represents the degradation rate constant, and t represents the number of release days.

#### 3.5.2. Storage Stability of ACN-NLs, PPI-ACN-NLs and SY-PPI-ACN-NLs Under Light

The stability of ACN-NLs, PPI-ACN-NLs and SY-PPI-ACN-NLs was examined in both light and dark conditions. Twenty milliliters of each compound was placed in four glass bottles, which were exposed to light or dark environments and stored at room temperature (25 °C) for 30 days, with samples collected every 5 days [57]. Observation and characterization were conducted, with the release rate of ACNs as described in Section 3.3.1, while MDA content was determined according to Section 3.3.6. Furthermore, zero-order and first-order degradation kinetics of the ACNs in the food simulation system were modeled, with specific release kinetics equations presented in Table 4.

#### 3.5.3. Storage Stability of ACN-NLs, PPI-ACN-NLs and SY-PPI-ACN-NLs in the Food Simulation System

The stability of ACN-NLs, PPI-ACN-NLs and SY-PPI-ACN-NLs was investigated in various food simulation systems. Four food simulants were chosen: a hydrophilic system (10% C_2_H_5_OH), an alcohol system (20% C_2_H_5_OH*)*, a lipophilic system (50% C_2_H_5_OH) and an alcoholic system (3% CH_3_COOH) [64]. Samples were stored in different food simulation systems at a 1:1 (*v*/*v*) ratio for 30 days, with samples taken every 5 days for observation and characterization. The release rate of ACNs was determined according to Section 3.3.1 and MDA was determined according to Section 3.3.6. Furthermore, the zero-order and first-order degradation kinetics of ACNs in the food simulation system were simulated, with specific release kinetics equations presented in Table 5.

## 4. Conclusions

In this experiment, ACN-NLs, PPI-ACN-NLs and SY-PPI-ACN-NLs with high encapsulation efficiency, small particle size and high absolute zeta potential were successfully prepared. Meanwhile, the changes in crystallinity were reflected by the peak shape alterations in X-ray diffraction (XRD) analysis. These results confirmed the successful modification of ACN-NLs into PPI-ACN-NLs and SY-PPI-ACN-NLs by SY and PPI. During in vitro digestion, the stability of SY-PPI-ACN-NLs was found to be higher than that of PPI-ACN-NLs, suggesting that the modified liposomes exhibited superior digestive stability under in vitro conditions. Therefore, it is recommended that liposomes be stored at 5 °C, shielded from light, and kept away from potassium ion-rich environments. Additionally, during product development, 10% ethanol systems should be considered whenever possible. Based on the results of this study, it can be concluded that in the food processing process, the use of SY-PPI modified nanoliposomes can enhance the stability of ACNs in high-temperature, ionic environments as well as in different food simulation systems, and can improve their utilization rate in sustained gastrointestinal release. The results of this study provide a reference for the application of SY-PPI double-layer-modified ACN nanoliposomes in the food, health care products and pharmaceutical industries, and have great potential in various food systems. Subsequent in vivo animal experiments will be conducted to investigate the bioavailability, targeted delivery capability, and metabolic mechanism of SY-PPI-modified nanoliposomes in complex physiological environments, and to clarify their actual sustained-release effects in the gastrointestinal tract and the pathways for exerting biological activity.

## Figures and Tables

**Table 1:** Effect of mass ratio of soybean lecithin to cholesterol on preparation of ACN-NLs.

Mass Ratio of Soybean Lecithin to Cholesterol (g/g)	Encapsulation Efficiency (%)	Particle Size (nm)	Zeta Potential (mV)
1:1	35.05 ± 0.22	198.67 ± 047	−12.20 ± 0.23
3:1	42.36 ± 0.33	138.77 ± 0.31	−24.8 ± 0.18
5:1	50.62 ± 0.43	128.70 ± 0.26	−38.23 ± 0.20
7:1	39.80 ± 0.25	114.17 ± 0.33	−35.97 ± 0.17
9:1	23.22 ± 0.41	103.30 ± 0.25	−32.37 ± 0.24

**Table 2:** Effect of mass ratio of soybean lecithin to anhydrous ethanol on preparation of ACN-NLs.

Mass Ratio of Soybean Lecithin to Anhydrous Ethanol (g/mL)	Encapsulation Efficiency (%)	Particle Size (nm)	Zeta Potential (mV)
1:100	47.41 ± 0.21	127.43 ± 0.23	−35.10 ± 0.24
2:100	48.35 ± 0.23	125.00 ± 0.15	−35.40 ± 0.10
3:100	51.59 ± 0.34	120.03 ± 0.33	−33.77 ± 0.21
4:100	48.77 ± 0.42	122.73 ± 0.28	−32.37 ± 0.34
5:100	42.91 ± 0.13	131.73 ± 0.31	−38.13 ± 0.27

**Table 3:** Influence of drug-to-lipid ratio on preparation of ACN-NLs.

Drug-to-Lipid Ratio (g/g)	Encapsulation Efficiency (%)	Particle Size (nm)	Zeta Potential (mV)
1:10	36.42 ± 0.45	81.46 ± 0.54	−34.10 ± 0.42
2:10	48.29 ± 0.25	135.30 ± 0.48	−34.43 ± 0.44
3:10	52.59 ± 0.34	134.13 ± 0.76	−32.40 ± 0.75
4:10	42.02 ± 0.48	150.43 ± 0.34	−26.77 ± 0.23
5:10	38.48 ± 0.15	262.73 ± 0.39	−22.63 ± 0.33

**Table 4:** Effect of pH value on preparation of PPI-ACN-NLs.

pH Value	Encapsulation Efficiency (%)	Particle Size (nm)	Zeta Potential (mV)
1	70.41 ± 0.17	244.43 ± 0.67	−12.40 ± 0.25
2	72.67 ± 0.22	174.73 ± 1.21	−29.63 ± 0.31
3	76.26 ± 0.14	159.50 ± 1.23	−34.06 ± 0.21
4	68.24 ± 0.15	157.40 ± 0.93	−33.31 ± 0.19
5	64.93 ± 0.18	152.80 ± 0.57	−23.36 ± 0.24

**Table 5:** Effect of PPI concentration on the preparation of PPI-ACN-NLs.

Pea Protein Isolate Concentration (mg/mL)	Encapsulation Efficiency (%)	Particle Size (nm)	Zeta Potential (mV)
4	74.43 ± 0.34	185.40 ± 0.27	−30.46 ± 0.54
6	78.20 ± 0.25	191.30 ± 034	−29.40 ± 0.48
8	83.80 ± 0.43	213.20 ± 0.41	−27.46 ± 0.69
10	83.40 ± 0.19	215.20 ± 0.23	−19.30 ± 0.21
12	83.10 ± 0.35	219.60 ± 0.18	−14.60 ± 0.12

**Table 6:** Effect of SY concentration on the preparation of SY-PPI-ACN-NLs.

Synanthrin Concentration (mg/mL)	Encapsulation Efficiency (%)	Particle Size (nm)	Zeta Potential (mV)
4	84.33 ± 0.38	224.66 ± 0.83	−16.76 ± 0.21
6	87.59 ± 0.44	241.00 ± 1.31	−16.79 ± 0.26
8	90.38 ± 0.24	246.60 ± 0.24	−16.93 ± 0.31
10	87.76 ± 0.22	251.16 ± 1.33	−16.32 ± 0.18
12	86.74 ± 0.50	263.40 ± 0.75	−17.16 ± 0.22

**Table 7:** Encapsulation efficiency, particle size and zeta potential of ACN-NLs, PPI-ACN-NLs and SY-PPI-ACN-NLs.

Sample	Encapsulation Efficiency (%)	Particle Size (nm)	Zeta Potential (mV)
ACN-NLs	52.59 ± 0.24	134.60 ± 0.76	−32.40 ± 0.75
PPI-ACN-NLs	83.80 ± 0.43	213.20 ± 0.41	−27.46 ± 0.69
SY-PPI-ACN-NLs	90.38 ± 0.24	246.60 ± 0.24	−16.93 ± 0.31

**Table 8:** Degradation kinetics of ACN-NLs, PPI-ACN-NLs and SY-PPI-ACN-NLs at different temperatures.

Sample		Zero Order			First-Order		
		R^2^	K	t_1/2(day)_	R^2^	K	t_1/2_
5 °C	ACN-NLs	0.78	0.52	1.33	0.99	0.09	7.57
	PPI-ACN-NLs	0.71	0.45	1.54	0.92	0.09	8.06
	SY-PPI-ACN-NLs	0.68	0.34	2.02	0.89	0.07	9.00
25 °C	ACN-NLs	0.78	0.88	0.79	0.95	0.11	6.36
	PPI-ACN-NLs	0.79	0.73	0.95	0.96	0.10	6.75
	SY-PPI-ACN-NLs	0.68	0.58	1.2	0.89	0.09	7.30
45 °C	ACN-NLs	0.80	1.49	0.47	0.94	0.13	5.51
	PPI-ACN-NLs	0.76	1.25	0.55	0.94	0.12	5.74
	SY-PPI-ACN-NLs	0.82	1.04	0.67	0.97	0.11	6.04
65 °C	ACN-NLs	0.88	2.09	0.33	0.99	0.14	5.02
	PPI-ACN-NLs	0.79	1.46	0.48	0.95	0.13	5.51
	SY-PPI-ACN-NLs	0.83	1.31	0.53	0.97	0.97	5.66

**Table 9:** Degradation kinetics of ACN-NLs, PPI- ACN-NLs and SY-PPI- ACN-NLs under light and dark conditions.

Sample		Zero Order			First-Order		
		R^2^	K	t_1/2(day)_	R^2^	K	t_1/2_
Illuminated	ACN-NLs	0.86	1.24	0.56	0.99	0.12	5.75
	PPI-ACN-NLs	0.79	0.95	0.73	0.96	0.11	6.22
	SY-PPI-ACN-NLs	0.75	0.79	0.87	0.92	0.11	6.56
Without light	ACN-NLs	0.78	0.88	0.79	0.95	0.11	6.36
	PPI-ACN-NLs	0.80	0.73	0.95	0.98	0.10	6.75
	SY-PPI-ACN-NLs	0.69	0.58	1.20	0.89	0.09	7.29

**Table 10:** Degradation kinetics analysis of ACN-NLs, PPI-ACN-NLs and SY-PPI-ACN-NLs.

Sample		Zero Order			First-Order		
		R^2^	K	t_1/2(day)_	R^2^	K	t_1/2_
10% C_2_H_5_OH	ACN-NLs	0.92	1.29	0.76	0.99	0.12	5.68
	PPI-ACN-NLs	0.97	0.77	0.89	0.98	0.10	6.93
	SY-PPI-ACN-NLs	0.94	0.48	1.45	0.94	0.09	7.87
20% C_2_H_5_OH	ACN-NLs	0.96	1.31	0.53	0.96	0.12	5.77
	PPI-ACN-NLs	0.98	0.78	0.88	0.98	0.11	6.33
	SY-PPI-ACN-NLs	0.91	0.47	1.46	0.99	0.09	7.79
50% C_2_H_5_OH	ACN-NLs	0.99	1.53	0.45	0.96	0.13	5.45
	PPI-ACN-NLs	0.96	1.02	0.68	0.98	0.11	6.08
	SY-PPI-ACN-NLs	0.93	0.61	1.13	0.99	0.09	7.14
3% CH_3_COOH	ACN-NLs	0.97	1.59	0.43	0.98	0.13	5.37
	PPI-ACN-NLs	0.961	1.24	0.55	0.98	0.12	5.72
	SY-PPI-ACN-NLs	0.89	0.88	0.78	0.99	0.11	6.35

## Data Availability

The original contributions presented in this study are included in the article/Supplementary Materials. Further inquiries can be directed to the corresponding authors.

## Supplementary Materials

The following supporting information can be downloaded at: https://www.mdpi.com/article/10.3390/molecules30142892/s1, Table S1: Results of Orthogonal Experiment for Preparation of ACN-NLs.; Figure S1: Appearance of ACN-NLs(A), PPI-ACN-NLs(B), and SY-PPI-ACN-NLs(C); Figure S2: Appearance of ACN-NLs, PPI-ACN-NLs and SY-PPI-ACN-NLs at different temperatures during storage; Figure S3: The release rate of ACNs and MDA values of ACN-NLs, PPI-ACN-NLs and SY-PPI-ACN-NLs during storage at different temperatures; Figure S4: Appearance of ACN-NLs, PPI-ACN-NLs and SY-PPI-ACN-NLs under light and dark conditions during storage; Figure S5: The release rate of ACNs and MDA values of ACN-NLs, PPI-ACN-NLs and SY-PPI-ACN-NLs under light and dark condition; Figure S6: Storage appearances of ACN-NLs, PPI-ACN-NLs and SY-PPI-ACN-NLs under different food simulations; Figure S7: The release rate of ACNs and MDA values of ACN-NLs, PPI-ACN-NLs and SY-PPI-ACN-NLs in food simulation system.
