# Supplementary material for: Preparation, Physicochemical Properties and Stability of Anthocyanin Nanoliposomes Before and After Double-Layer Modification Using Synanthrin and Pea Protein Isolate
Source: Molecules. 2025 Jul 8;30(14):2892. doi: 10.3390/molecules30142892 (PMC12297960; doi:10.3390/molecules30142892)
Supplement: Supplementary file 1 [file molecules-30-02892-s001.zip › molecules-3682132-supplementary.pdf]

Supplementary

Table S1. Results of Orthogonal Experiment for Preparation of ACN-NLs

Test number	(A) Mass Ratio of Soybean Lecithin to Cholesterol	(B) Mass Ratio of Soybean Lecithin to Anhydrous Ethanol	(C) Ratio of drug to lipid	Encapsulation efficiency (%)	Particle size (nm)	Zeta potential (mV)
1	1	1	1	46.53±0.24	147.00±0.76	-28.30±0.26
2	2	2	2	52.59±0.31	134.60±0.65	-34.50±0.17
3	3	3	3	42.12±0.14	144.20±0.63	-32.56±0.11
4	1	2	3	39.40±0.38	147.86±0.71	-28.70±0.23
5	2	3	1	45.10±0.29	115.80±0.19	-29.43±0.08
6	3	1	2	42.09±0.43	162.30±0.27	-22.13±0.36
7	1	3	2	40.86±0.17	157.17±0.54	-27.20±0.45
8	2	1	3	34.70±0.48	165.47±0.81	-18.80±0.05
9	3	3	1	43.90±0.51	149.17±0.26	-34.00±0.19
K_1	126.79	123.32	135.53			
K_2	132.39	135.89	135.54			
K_3	128.11	128.08	116.22			
R	5.60	12.57	19.32			

Note: K_1, K_2 and K_3 are the total values of the encapsulation efficiency under factors A, B, and C, respectively; K_1, K_2, and K_3 are the total values; R is the range.

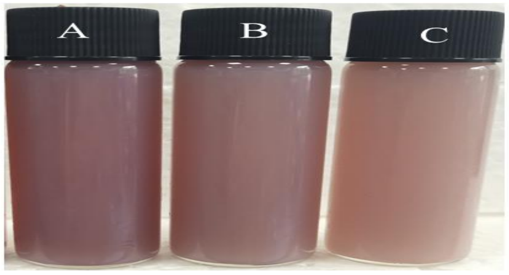

Figure S1. Appearance of ACN-NLs(A), PPI-ACN-NLs(B), and SY-PPI -ACN-NLs(C)

Note: Anthocyanins (ACNs), ACN-NLs (anthocyanin nanoliposomes), PPI-ACN-NLs (Pea Protein Isolate modified anthocyanin nanoliposomes), SY-PPI-ACN-NLs (synanthrin, Pea Protein Isolate modified anthocyanin nanoliposomes)

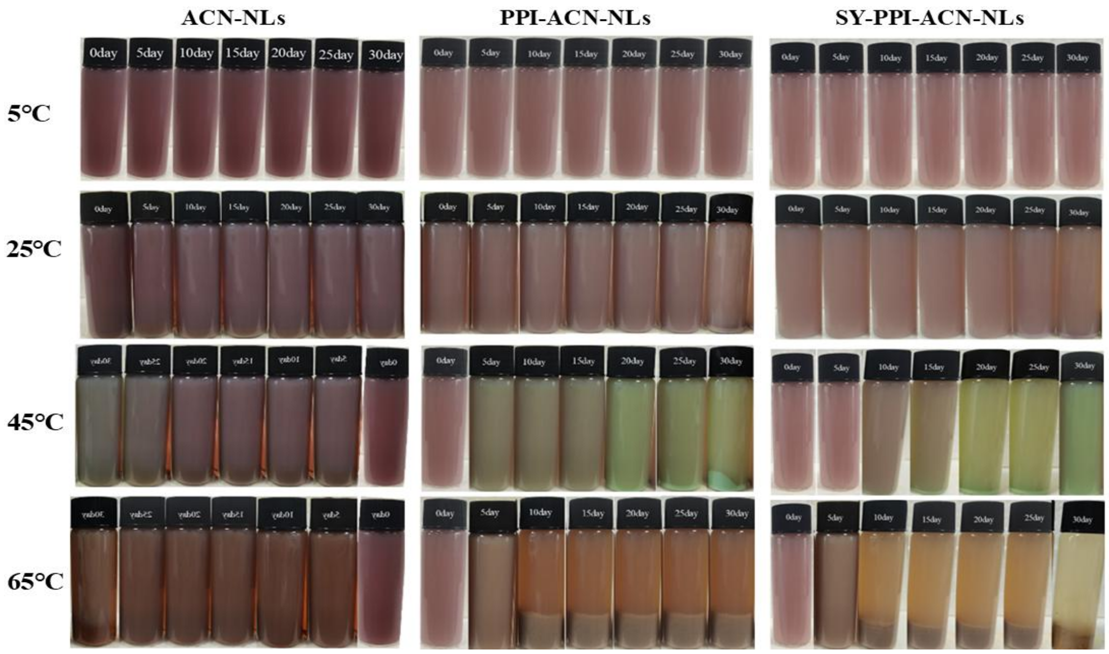

Figure S2. Appearance of ACN-NLs, PPI-ACN-NLs and SY-PPI -ACN-NLs at different temperatures during storage

Note: Anthocyanins (ACNs), ACN-NLs (anthocyanin nanoliposomes), PPI-ACN-NLs (Pea Protein Isolate modified anthocyanin nanoliposomes), SY-PPI-ACN-NLs (synanthrin, Pea Protein Isolate modified anthocyanin nanoliposomes)

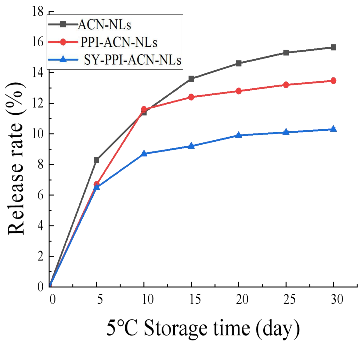

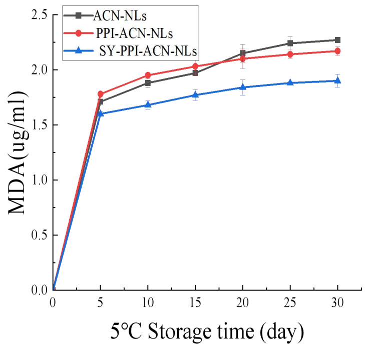

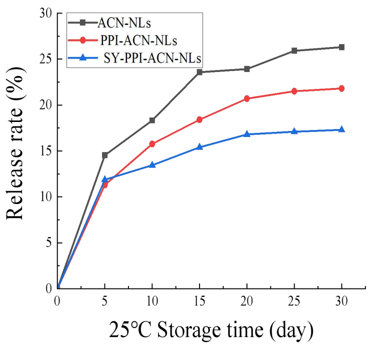

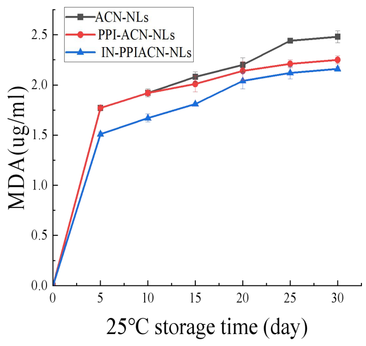

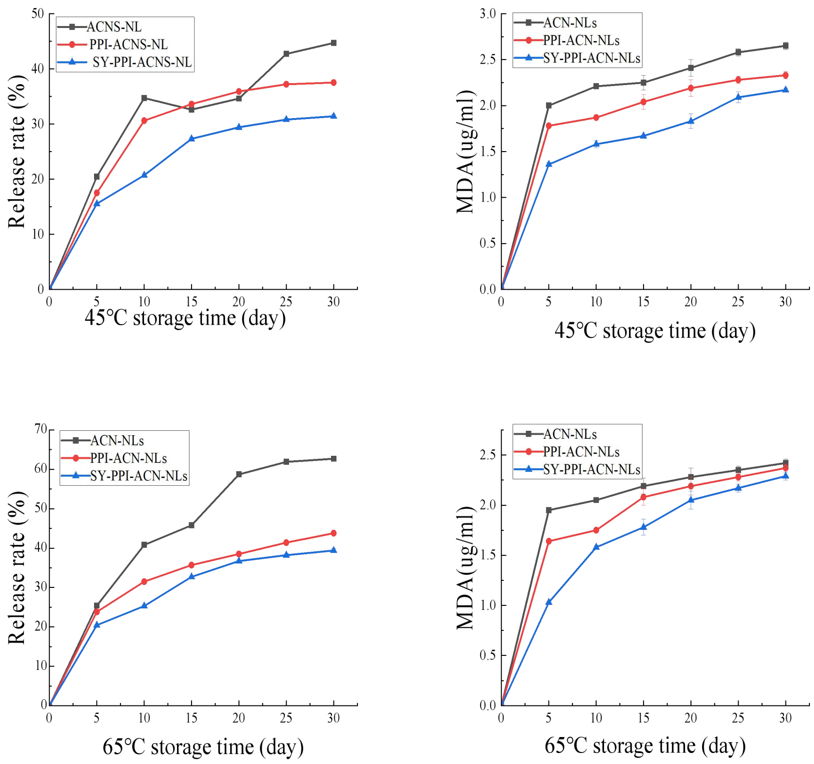

Figure S3. The release rate of ACNs and MDA values of ACN-NLs, PPI-ACN-NLs and SY-PPI-ACN-NLs during storage at different temperatures

Note: Anthocyanins (ACNs), ACN-NLs (anthocyanin nano-liposomes), PPI-ACN-NLs (Pea Protein Isolate modified anthocyanin nano-liposomes), SY-PPI-ACN-NLs (synanthrin, Pea Protein Isolate modified anthocyanin nano-liposomes); MDA values (malondialdehyde values)

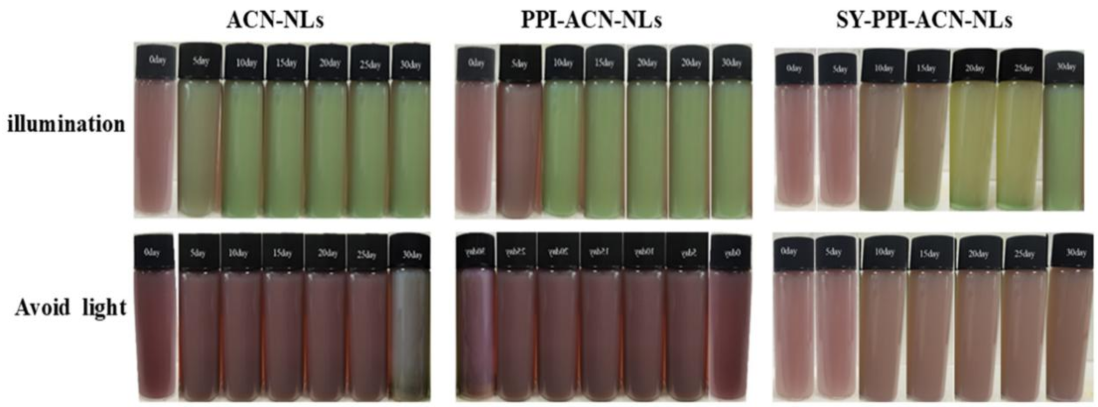

Figure S4. Appearance of ACN-NLs, PPI-ACN-NLs and SY-PPI-ACN-NLs under light and dark conditions during storage

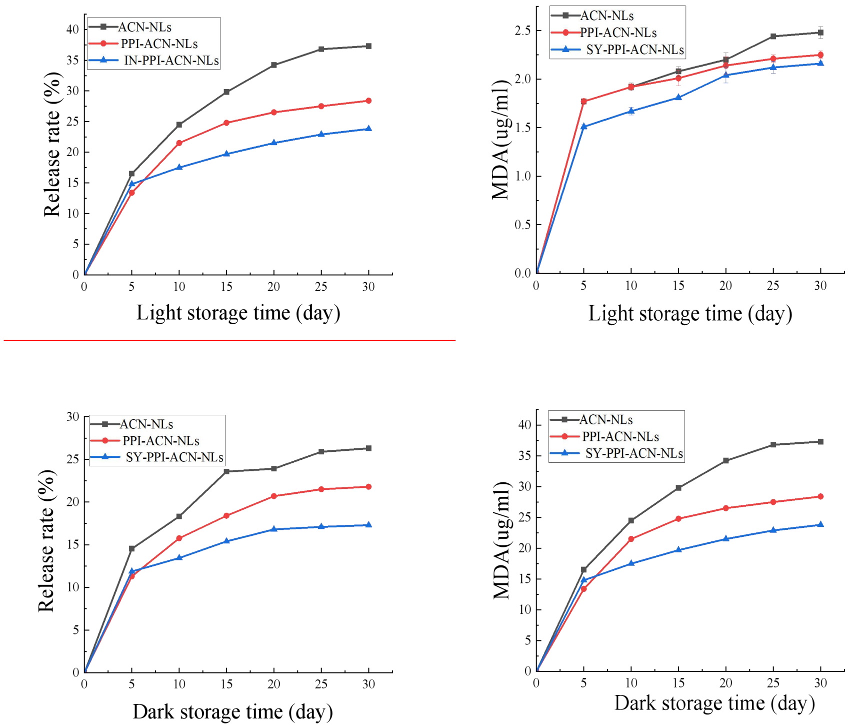

Figure S5. The release rate of ACNs and MDA values of ACN-NLs, PPI- ACN-NLs and SY-PPI-ACN-NLs under light and dark condition

Note: Anthocyanins (ACNs), ACN-NLs (anthocyanin nano-liposomes), PPI-ACN-NLs (Pea Protein Isolate modified anthocyanin nano-liposomes), SY-PPI-ACN-NLs (synanthrin, Pea Protein Isolate modified anthocyanin nano-liposomes); MDA values (malondialdehyde values)

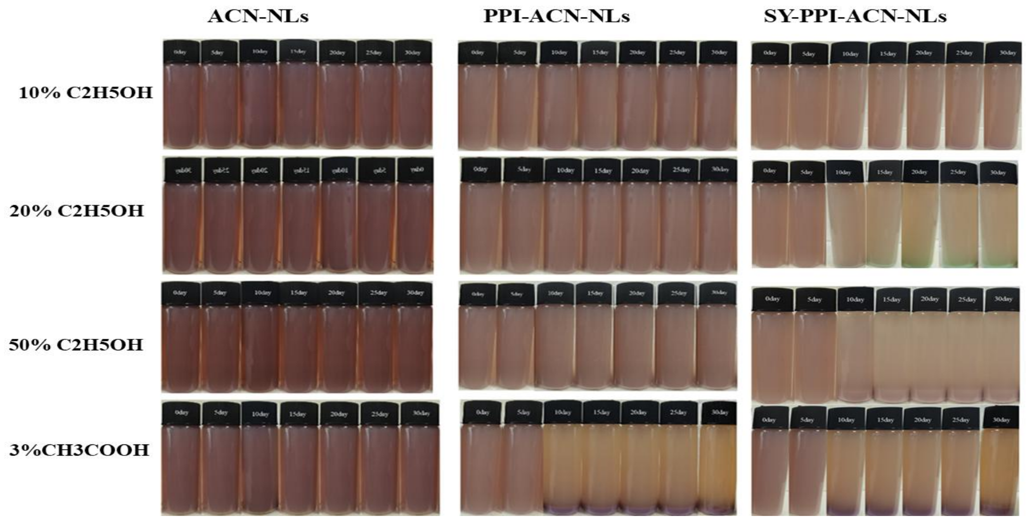

Figure S6. Storage appearances of ACN-NLs, PPI-ACN-NLs and SY-PPI-ACN-NLs under different food simulations

Note: Anthocyanins (ACNs), ACN-NLs (anthocyanin nano-liposomes), PPI-ACN-NLs (Pea Protein Isolate modified anthocyanin nano-liposomes), SY-PPI-ACN-NLs (synanthrin, Pea Protein Isolate modified anthocyanin nano-liposomes); MDA values (malondialdehyde values)

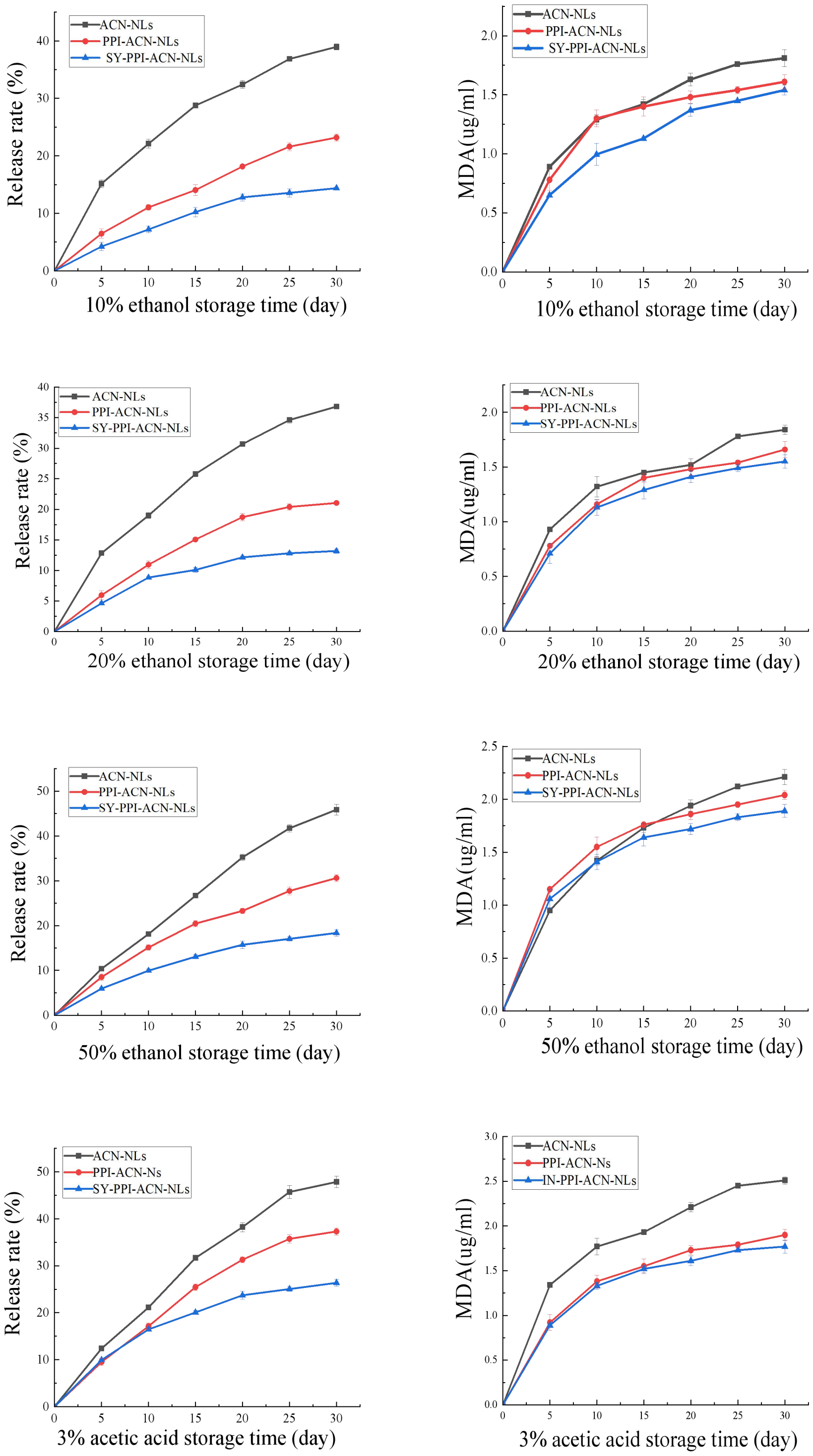

Figure S7. The release rate of ACNs and MDA values of ACN-NLs, PPI-ACN-NLs and SY-PPI-ACN-NLs in food simulation system

Note: Anthocyanins (ACNs), ACN-NLs (anthocyanin nano-liposomes), PPI-ACN-NLs (Pea Protein Isolate modified anthocyanin nano-liposomes), SY-PPI-ACN-NLs (synanthrin, Pea Protein Isolate modified anthocyanin nano-liposomes); MDA values (malondialdehyde values)
